# The Essential Factors of Establishing Patient-derived Tumor Model

**DOI:** 10.7150/jca.51749

**Published:** 2021-01-01

**Authors:** Chuanzhi Chen, Wu Lin, Yingying Huang, Xiangliu Chen, Haohao Wang, Lisong Teng

**Affiliations:** Department of Surgical Oncology, the First Affiliated Hospital, School of Medicine, Zhejiang University, Hangzhou 310003, China

**Keywords:** patient-derived xenograft, take rate, engraftment rate, tumor burden, Hormonal supplements, Humanized mouse

## Abstract

Establishing an applicable preclinical model is vital for translational cancer research. Patient-derived xenograft has been important preclinical model systems and widely used for cancer research. Patient-derived xenograft models that represent the tumors of the patients are necessary to better translate research discoveries and to test potential therapeutic approaches. However, research in this field is hampered by the limited engraftment rate. In this review, we go over a large number of researches on patient-derived xenograft transplantation and firstly systematically summarize the main factors in methodology to successfully establish models. These results will be applied to the development of patient-derived xenograft leading to better preclinical research.

## Background

Cancer is a major threat to human health and its morbidity keep rising in recent years. Meanwhile, cancer is one of major cause of death in men^1^, including lung, breast, gastric and colorectal cancer. Over the past decade, with the developments in surgery, chemotherapy and targeted therapies, the prognosis of certain malignancies has improved greatly. Patients diagnosed with early stage cancer have high cure rate, but the advanced cancer still result in a poor survival rate^2^. Due to the complexity and heterogeneity of the tumor, novel techniques and research tools is indispensable for developing personalized and targeted treatments. Preclinical models are needed to identify and test new precision cancer therapies.

**Figure 1:**
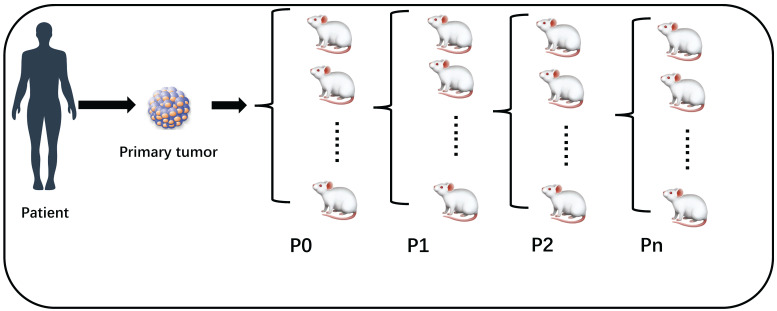
The success rate of P0 is take rate, engraftment rate is calculated at least from P2.

Patient derived xenografts (PDX) hold significant promise for establishing preclinical model, which is immunodeficient mice engrafted with patient tumor tissue. Recent researches have shown PDX can closely retain genetic, phenotypic and histopathologic features of the original tumors^3^-^5^. Therefore, PDX apply to validate biomarkers of response and test the efficacy of novel therapies. From these preclinical models to clinic, translating experimental results will promote the identification of drug-sensitive patients and guide therapy selection. Generally, PDX engrafted from patient tumor specimens to mouse was P0, then derived into generation 1 (P1), generation 2 (P2), etc. (Figure 1) Technically, engraftment rate was defined as successfully grew through at least 3 serial passages of the PDX model, i.e., starting from P2. Some studies have confused this concept by using take rate (P0) as engraftment rate, this is not accurate. In our table, we mark the 'Passage' column to distinguish this difference (Table 1). However, according to the previous studies, Engraftment rate of PDX models are inefficient^6^-^8^. Tumor samples and given cell lines fail to grow tumors at graft sites for various factors. It's particularly difficult to passage first generation Xenograft model to second generation. Low engraftment rate hampers the preclinical model translating drug-response into clinical practice, incapable guiding oncologists to select the best targeted therapy.

Our team has long been committed to the establishment and application of PDX model for simulating human tumor tissue. In the past 10 years, based on the international modeling theory and practice of our team, a standardized method and technical system for surgical sampling, specimen transfer, transplantation and tumor inoculation, cryopreservation, resuscitation has been established. Our PDX model show good consistency with the primary tumor tissue 9, 10 . In this review, we went over the most recent advances of preclinical studies and basic research in which PDX have been used. Combining with our current methodology for the generation of PDX models, so as to summarize the key factors to establish PDX models. We envision that, as these points are handled properly, PDX will be constructed more effectively, which in turn provide more potentially predictive value.

## Tumor burden

According to National Cancer Institute (NCI), tumor burden refers to the number of cancer cells, the size of a tumor, or the amount of cancer in the body 11. Whether the PDX model can be established is tightly related to the tumor burden of the patients. Tumor burden can not only reflect the degree of tumor activity, but also reflect the drug response. At the same time, from the perspective of molecular level, tumor burden even can reflect the progress trend of cancer cells and tiny cancer tissues^12^. Measuring tumor burden as a method provide ability to differentiate between primary and metastatic tumors. In pancreatic cancer, tumor burden has better predictive performance for both overall survival (OS) and recurrence-free survival (RFS) than serum CA199 levels^13^. One study indicated that suppressing tumor burden with small molecule inhibitor can hinder the growth of subcutaneous transplanted tumor cells^14^. The efficiency of PDX model establishment was highly correlated with the tumor burden of patients when sample was removed. The higher tumor burden result in the higher engraftment rate. All the selection of patients, sampling site, sampling size and sampling time have great influence on the PDX construction.

### Tumor stage

Chen Y et al. found different tumor stage play a vital role in engraftment rate which can roughly reflect tumor burden. In non-small cell lung cancer, tumor samples from patients with stage II (43/96, 45%) and stage III (25/49, 51%) disease showed relatively high engraftment rates than stage I (32/145, 22%) 15. Oh et al. also confirm that advanced stage tumor tends to have significantly higher tumor take rates in colorectal cancer xenograft mice. Their results show that xenografts established from 4 of 15 (26.7%) stage I tumors, 41 of 72 (56.9%) stage II, 50 of 84 (59.5%) stage III tumors, and 55 of 70 (78.6%) stage IV tumors^16^. Moreover, these hepatocellular carcinoma sample removed from patients with large size tumor (>5cm) have higher PDX establishment rate (87/130, 67%) compare with small size (≤5cm) (16/124, 12.9%)^17^. Similarly, Jung et al. successfully produced 20 PDXs of pancreatic cancer, they found that tumor size is a significant factor of the success of PDX^18^. Similarly, their another study suggest that N stage is a clinical factor affecting PDX derivation^19^. Weroha et al. Successfully engrafted 124 ovarian cancer models with a 74% engraftment rate and affirmed that successful mouse engraftment correlated with adverse patient characteristics such as advanced stage, presence of ascites and high-grade tumors^20^. Moreover, in squamous cell head and neck cancer, the average survival of patients whose PDX engrafted was 21.1 months in comparison to no engraftment with 28.4 months, suggesting a relatively short survival if the tumor grew on mice^21^. Later tumor stage indicates a poor prognosis for overall survival (OS) and disease-free survival (DFS), that is to say, tumor cells are more aggressive and metastatic, which contribute to engraft successfully. Another research showed PDX models could be successfully created from clinical biopsy specimens in patients with metastatic or unresectable gastrointestinal cancers^22^, ^23^. When it comes to scientific research, choosing samples with high tumor stage is helpful to establish a better PDX model. In addition, metastatic tissue could be a preferable choice for drug screening experiments.

### Metastatic sample

Researches have shown metastatic cancers exhibit higher PDX model engraftment rates compared to nonmetastatic cancers^22^, ^24^-^26^. Masanori and colleagues generated PDXs model of human brain metastases of breast cancer in the mouse brain. This method had no perioperative mortality and a 100% (10/10) engraftment rate^27^. In a colon cancer PDX research, 100% (8/8) engraftment rate were achieved with metastases compare with the 84% (27/32) engraftment rate with primary cancer^28^. Remarkably, the engraftment rate of tumors implanted from metastases was relatively higher than that of primary tumors. Higher engraftment rates were also observed with samples implanted from primary tumors with distant metastases^29^. Similarly, 16 patients underwent potentially curative resection of colorectal liver metastases, and tumors were grafted into immunodeficient mice. Overall, 81% engraftment rate was achieved. Moreover, there was a 67% positive match rate between applicable patient and PDX chemosensitivity profiles^30^. These data suggested that the capability of tumors to grow serially in mice was associated with their capability to metastasize and seed distant sites. And the growth rate of metastases is not as limiting. In some cases, PDX models indeed demonstrated the genomic and transcriptomic signature of metastatic and relapsed cancers^24^. We believed that the probability of engraftment is higher when samples are obtained from metastases relative to primary tumors.

### Treatment status

Whether patients receiving treatment before tumor resection will impede the successful establishment of the PDX model remains controversial. In a research of non-small cell lung cancer PDX, 32% (81/247) engraftment rate were achieved without preoperative chemotherapy compare with the 37.3% (22/59) in chemotherapy group. Therefore, Preoperative chemotherapy was found to have no significant effect on non-small cell lung cancer engraftment rates^15^. Notably, in a recent study, samples from 133 patients with resected pancreatic ductal adenocarcinoma were engraft in mouse. As a result, 42 (32%) patients in their series received neoadjuvant chemotherapy while 91 (68%) patients without neoadjuvant chemotherapy and this did not adversely affect tumor engraftment^31^. Partly because patients requiring neoadjuvant chemotherapy have a high grade of tumor, and even after chemotherapy, their malignancy, still be aggressive and metastatic, which makes no difference in implantation rate compared with patients who do not need chemotherapy.

Based on the accessible data, we cannot conclude that the administration of chemotherapy will affect the successful establishment of PDX.

### Tumor type and subtypes

Among various tumors, breast cancer has the relatively lowest success rate of PDX engraftment, ranging from 21% to 37% in various studies (Table 1). Since breast cancer is a hormone dependent disease, hormonal receptor status determines the treatment regimen. Moreover, immunodeficiency mice can't provide the hormones needed for tumor growth after transplantation tissue engrafted from human body to mice, which leads to the difficulty in establishing breast cancer PDX model. Thus, transplantation rate for triple negative breast cancer is relatively higher than other breast cancer types. In human breast cancer, tripe-negative breast cancer yielded the highest take rate (51.3%), followed by HER2+ (26.5%) and luminal B (5.0%). Moreover, the stable take rate of ER negative (52%) and PR negative (37%) tumors was noticeably higher than that of ER positive (2%) and PR positive (3%) tumors^32^. Due to cells in different tissues varying greatly, often multifocal, establishing the xenograft of prostate cancer cells which can show the original tumor characteristic is pretty difficult, and prostate cancers rarely grow as xenografts^33^. On the other hand, Colorectal cancer, Pancreatic cancer, Head and neck cancer and Ovarian cancer show acceptable engraftment rate in immunodeficiency mice. Interestingly, a novel research collected tumor tissue samples from 308 patients who were diagnosed with non-small cell lung cancer and implanted in immunodeficient mice, which revealed squamous cell carcinomas had a higher engraftment rate compared with adenocarcinomas^15^, ^34^. Undifferentiated tumors have aggressive phenotype with a potential to give rise to metastatic growth after implantation of a few tumor cells, nevertheless well differentiated tumors are found to be much less aggressive^20^, ^35^.

## Materials handling

### Sample volume

Generally, choosing appropriate tumor volume for implantation improves the engraftment rate of PDX model. Comparatively large tumor volume will easily affect the accuracy of tumor transplantation operation, leading to the deviation of engrafting position and reducing the transplantation rate. In addition, tumor tissue is pretty limited and precious, in order to establish sufficient number of models for a patient, the implant volume of each host should not be oversize. Undersized tumor may not fully reflect the heterogeneity of the primary tumor, consequently, influencing the prediction value of the PDX for drug-screening. As a matter of fact, we recommend that the tumor mass could be minced into 2×2×3 mm^3^ fragments after removing the overlying capsule connective tissue. It has been suggested that isolated cells can increase the rate of tumor transplantation in the study of Anderson et al. Tumor Samples were gained by endobronchial ultrasound-guided transbronchial needle aspiration, then isolated cells from the aspiration samples were subcutaneously implanted into recipient mice. Their result showed 67% patient samples produced confirmed small cell lung cancer tumors^36^. Interestingly, Drapkin et al. use circulating tumor cells as an emerging source of cancer cells for establishing small cell lung cancer PDXs, which showed efficiency in representing patient tumor characteristics, including genomic, transcriptional profiles and drug sensitivities. However, xenografts generated from biopsy are more effective than isolated cells or circulating tumor cells with 89% engraftment rate^37^. Moreover, isolated cells may not fully represent the cellular diversity of a patient tumor. Therefore, it must be stressed that the use of isolated cells will affect the histological structure and matrix secretion of the tumor, thus affecting the heterogeneity of the tumor. According to the research data, most PDX models were derived from the engraftment of tissue fragments rather than isolated cells^38^. In terms of maintaining the heterogeneous nature of human cancers, indeed, spatial variation exists in a tumor's clonal composition. The existence of multiple subclones explains variable response rates to precise treatment, even within a single tumor mass, and rapidly emerging of drug resistance^39^. Therefore, implantation sample should better not be too diminutive. All in all, suitable specimen size is one of the most basal factors for successful transplantation.

### Surgery

Remarkably, different surgical procedures tend to produce samples that are more or less suitable for growing PDXs. For malignant tumors, radical surgical resection is superior than partial resection or palliative resection in preserving the integrity of the tumor. This can be confirmed in the comparison of Transurethral resection of the prostate (TURP) with radical prostatectomy for prostate cancer. In a prostate cancer study, tissue was derived from radical prostatectomy in 25 patients and from palliative TURP in 2. And their results show all growing PDX model developed from tissue derived from radical prostatectomy^40^. Another research indicated that their TURP tissue (I) was at least 50% cancer cells, (II) had no physical damage, and (III) had detectable Ki67 expression, which was the easiest to transplant successfully. However, only 21% grafts contained cancer at harvest^41^. It suggested that the TURP is more prone to generating tissue fragments, resulting in the destruction of tissue structure, thereby reducing tumor heterogeneity and decreasing the invasion of the tumor cells. Moreover, Katsiampoura et al. found that the PDX development success rate was higher in surgical (36/50 = 72%) than biopsy (14/40 = 35%) specimens in colorectal cancer. Generally, a specimen removed by radical surgery will be more preferable^42^. But in some unresectable cases, in order to open up xenografting to a wider cancer patient population, multiple-spots aspiration was applied to obtain the specimens^43^.Therefore, using biopsy specimens for PDX transplantation is an optional approach as well. By the way, the process of sample collection, preservation and transportation are crucial, which ensure maximum freshness of the sample. In summary, the surgical methods of specimens should be selected as completely as possible when the PDX model is established, including radical surgery and majority resection.

### Hormonal supplements

In the establishment of hormone-dependent cancer PDX models, the addition of hormones is contributing to simulating the primary tumor microenvironment in human body, thus improving the efficiency of PDX model engraftment. Considering the critical role of tumor microenvironment in tumor progression, cellular interactions with the hormone secretion can slightly alter gene expression programs, drive differentiation and profoundly alter cell biological behavior^4^. Therefore, it is helpful to preserve the tissue structure of the tumor as much as possible to successfully transplant the tumor into mice. In a study of the PDX modeling microenvironment for breast cancer, the researchers established three conditions: Condition 1: unmanipulated host mice, Condition 2: Estradiol supplementation and Condition 3: Estradiol and human fibroblasts supplementation. Primary tumor fragments were transplanted directly into epithelium-free “cleared” fat pads of recipient mice with corresponding condition. Of the conditions tested, xenograft take rate was highest under transplantation condition 2, with the underlying assumption that the presence of a low-dose estradiol pellet^44^. The study demonstrated that the addition of hormones can increase the rate of tumor transplantation. Moreover, supplementation with exogenous androgens shortened the latent period of tumorigenesis and displayed faster tumor growth. In Wu et al.'s research, Control group was subcutaneously implanted with the tissue without testosterone, whereas in the testosterone groups implantation was performed subcutaneously or under the renal capsule and testosterone was supplemented. As a result, mice supplemented with androgen displayed faster tumor growth than those in the control group^45^. Thus, during the establishment of PDX in hormone-dependent tumors, the addition of a certain amount of the corresponding hormone in mice will help to derive the tumor more efficiently.

### Harvested volume

After primary engraftment tumor growth, tumor was inoculated into mice to develop next passages of PDX. The optimum of tumor harvested volume upon engraftment remains controversial in PDX models. Although the experimental procedure of previous studies shows differences, the harvest time is usually determined by assessing the tumor volume. As Zhang and his colleagues highlighted, Regardless of the source of tumor cells, when primary transplantation reached 1000mm^3^, fragments were re-transplanted into new hosts as secondary xenografts^44^. Another team who achieved 90% engraftment rate have empirically determined that it would require 2000mm^3^ of tumor tissue 46. Moreover, a large sample on colorectal cancer suggest mice were euthanized when tumor was reaching 1500mm^3^^42^. In a head and neck cancer research, tumors were harvested when larger masses for a given PDX approximately reached 1000mm^3^^47^. Large tumors easily affect the survival state of mice, causing the lack of nutrients of tumor cells and the weakening of tumor transplantation capability. Moreover, the tumor growth curve is close to the maximum value, with low time utilization ratio and the nearly flat curve. Focusing on time-efficient engraftment for purpose of seeing a benefit within a clinically appropriate timescale. If the harvested tumor was undersize, there would be inadequate heterogeneous cells to form the next generation of tumor transplantation, leading to the failure of transplantation or the inability to represent the drug sensitivity of the primary tumor. Basing on the above data and combining our experience in the PDX model of colorectal cancer, we believe that the best harvested volume for transplantation is 1000-2000mm^3^. Furthermore, we should also give priority to the time from implantation of the tumor to removal. The existence of no tumor growth state requires us to set a deadline. If the tumor has not reached the ideal transplant size, beyond the cut off time, mice also have to be euthanized. Commonly, no overt tumor formation was observed by 30 weeks, tissues should be harvested and processed for histological evaluation. In conclusion, combining suitable harvested size and terminal time for PDX cancer research can improve the efficiency of preclinical research.

### Site of implantation

Subcutaneous transplantation rates were low, except that subrenal capsule implantation increased the rate of non-small cell lung carcinoma (NSCLC) implantation. Over the years, it has become increasingly clear that engraft in subrenal capsule of mice achieved a high engraftment rate. Subrenal capsule as an enveloping membrane environment which is similar to the lung membrane and secretes glucocorticoids, promotes the growth of lung cancer tissues. Importantly, previous research indicated that tumor-promoting effect is mediated via the plasma membrane integrin αvβ3 and increased lung cancer neoangiogenesis^48^. Moreover, the most common engraftment site for prostate cancer models is subrenal, which shows a high implantation rate (Table 1). Some studies have shown that the transplantation rate of orthotopic transplantation is higher than that of subcutaneous transplantation, which makes orthotopic transplantation a preferred method for the modeling of cancer^18^. Nevertheless, orthotopic site have demonstrated limited engraftment success rates in pancreatic cancer. Interestingly, Read et al.'s novel implantation technique whereby the tumor tissue is placed in a dorsal intramuscular pocket, successful engraftment was achieved for all patient tumors. Among these PDXs, 72% recapitulated the original patient tumors with respect to degree of differentiation, genetic and molecular profiles, and chemotherapeutic response^49^. This suggests intramuscular transplant technique cloud be a potential establishment approach for PDX in future clinical trials and clinical practice. In short, the selective location and environment of the transplantation site should depend on the specific biology of the tumor.

## Host strain

We recommend that different mouse strains, with different immune characteristics, should be used in diverse tumor. Selecting the appropriate host strain can help improve the efficiency of establishing PDX model. Typically, NK cells have been suggested to be involved in tumor xenograft rejections. According to the previous studies, the more frequently used mouse strains are: NOD/SCID, NSG, Balb/c nude, SCID mice. Firstly, the NOD/SCID mouse is commonly used for PDX models because it does not produce natural killer cells, meanwhile, with a high thymic lymphoma incidence. NSG has a null mutation in the gene encoding the interleukin-2 receptor gamma chain, long median survival (>89w), low lymphoma incidence, leading to dysfunctions of innate immunity including natural killer cell differentiation^19^, ^50^. Both the innate and acquired immune systems of NSG mouse are impaired. However, Balb/c nude has an innate immune system but no acquired immunity^51^. In some ways, SCID mice are excellent recipients of prostate xenografts, which show successful xenografting of mature blood cells and human hematopoietic stem cells^5^.

The efficiency of selection of mouse strains can be rendered in some studies. Dong et al. used NOD/SCID to establish non-small cell lung cancer PDX model with 90% engraftment rate, and examined for determining responses to conventional chemotherapeutic regimens^46^. Zhou and colleagues evaluated antitumor activity of salinomycin in the NOD/SCID Uveal melanoma xenograft model, and certificated NOD/SCID is effective host for melanoma preclinical research^52^. Likewise, Kimple et al. established head and neck cancer PDX with 85% engraftment rate via NSG. BLZ-100 can distinguish high-risk from low-risk dysplasia, through NSG PDX, which show as sensitive and specific marker of Squamous Cell Carcinoma of the Head and Neck (SCCHN)^53^, ^54^. Studies of long-chain non-coding RNA in ovarian cancer commonly use NSG mice as transplant hosts as well^55^, ^56^. In previous study, 15 colorectal carcinoma (CRC) PDX model were successfully established, with engraftment rate of 100%, using BALB/c nude as hosts^57^. Moreover, another research found 93% of xenografted pancreatic carcinoma engrafted satisfactorily in Balb/c nude^58^. This host strain has also been frequently applied in some especially central studies of gastric cancer. The IgG levels were significantly high in patients with gastric and colorectal cancer. Furthermore, elevated IgG level was associated with the advanced gastric cancer. The B lymphocytes in Balb/c mice were normal, but functionally deficient. The antibody was mainly IgM, with only a small amount of IgG. These partly explains why Balb/c nude a suitable host for gastrointestinal tumors^59^-^62^. Interestingly, Balb/c nude mice can be used for the isolation of populations of Human renal cell carcinoma (HRCC) cells with different growth and metastatic potential and that, of the organ sites tested, kidney, pancreas, seminal vesicles and lymph nodes are feasible for implantation of HRCC cells^63^. The subrenal capsule site in SCID mouse was found to be greatly efficient with nearly 95% of grafts recovered in prostate cancer^64^.

Overall, NOD/SCID mice was mostly used for lung cancer and melanoma, NSG mice for breast cancer, SCCHN and Ovarian cancer, Balb/c nude for colon cancer, Pancreatic carcinoma and Gastric cancer and Renal cell carcinoma, and SCID mice for prostate cancer. This suggests that in future, the host strain can be precisely selected depending on its xenograft type.

### Humanized mouse

However, conventional PDX models have limitations in studying immune-cancer interactions and preclinical evaluation of cancer immunotherapy, that is, they do not reflect the human immune system. To overcome these constraints, recent research has developed a technique to expand human hematopoietic stem and progenitor cells and use them to reconstitute the immune system in mouse, then transplant the patient's tumor into the mice, known as humanized Patient derived xenograft (Hu-PDX)^65^, ^66^. Hu-PDX is a critical tool for studying tumor immunity, inflammation, and infectious disease. With recent advances in human PDX, the take rate of P0 is encouraging. Capasso et al. established colorectal cancer microsatellite stable and microsatellite instable-high Hu-PDX, with tumor takes of 94% and 89% respectively^67^. In breast cancer, under the same experimental conditions, the positive rate of Hu-PDX was about 80-85%, slightly lower than that of non-humanized PDX (95-100%) 68. Moreover, Meraz and colleagues described the development of an improved Hu-PDX model that represent the human tumor microenvironment. Their results showed that approximately 60% to 80% of PDXs implanted in two different molecular types of Hu-PDX developed into tumors^69^. But current Hu-PDX model has several limitations: 1) the immune status and tumor microenvironment were not stable during Hu-PDX passage. 2) the balance of immune cells and hematopoietic were different in humans. 3) Hu-PDX is labor-intensive and time-consuming. As a preclinical model, these problems still require attention for the development of Hu-PDX model. In general, the Hu-PDX model includes not only patient-derived tumors, but also human hematopoietic stem cells to obtain full repertoire of human immune cells, which will be helpful for the further research of tumor immunotherapy.

## Conclusion

With the improvement of experiment protocol and scientific research conditions, the rate of PDX model establishment of most cancers has been greatly improved, but inefficiencies in the establishment of PDX in some cancers still hamper basic research and require further studies. To the best of our knowledge, this is the first review that systematically summarize the main factors in methodology to successfully establish PDX models. In our study, we discussed the role of engraftment site, host strain and cancer type in patient derived xenograft model. High stage and histological grade tumor tend to successfully transplant in immunocompromised mice. Commonly, the choice of mice species varies from the cancer species, so as the engraftment site. But subrenal capsule site transplantation show high engraftment rate in most kinds of cancer, such as Non-small cell lung carcinoma, Prostate cancer, Ovarian cancer et al. Moreover, we suggest that the tumor burden should be taken into consideration when selecting patients. Unfortunately, there is insufficient data to suggest that the treatment status of cancer patients at the time of specimen removal will influence the transplantation of specimens. After completely surgical excision, transplanting appropriate size of primary tumor tissue directly into mice allows for the resemblance of the tumor microenvironment. Interestingly, the addition of hormones in some tumors can help shorten the growth period of PDX and increase the engraftment rate. It is of high importance to combine the selection of specimens to process of engraftment and host strain, which will prompt more efficient translation from bench to bedside. With the development of reliable engraftment, PDX could lead to precise preclinical models for individual patients.

## Figures and Tables

**Table 1:** Summary of success rate of PDX models

Tumor type	Host strain	Implantation site	Take/Engraftment rate	Passage	reference
Breast cancer	NOD/SCID&NSG NOD/SCID NSG&SCID/Beige NOD/SCID Balb/c nu/nu	subcutaneous orthotopic orthotopic orthotopic subcutaneous	27% 37% 21% 35% 27%	P0 P0 P2 P0 P0	Jia et al.^70^ Derose et al.^71^ Zhang et al.^44^ Fiche et al.^72^ McAuliffe et al.^73^
Non-small cell lung carcinoma	NOD/SCID NOD/SCID&NSG SCID&nude SCID&nude	Renal capsules Subcutaneous Subcutaneous Subcutaneous	90% 34% 39% 35%	P0 P6 P2 P2	Dong et al.^46^ Chen et al.^15^ Moro et al.^74^ Llie et al.^34^
Colorectal carcinoma	Balb/c nude Balb/c nude Nude NOG NSG	Subcutaneous Subcutaneous Subcutaneous Subcutaneous Subcutaneous	62% 59% 54% 73% 72%	P0 P0 P0 P0 P0	Oh et al.^16^ Guan et al.^75^ Cybulska et al.^7^ Fujii et al.^76^ Katsiampoura et al.^42^
Prostate cancer	NOD/SCID NSG&NOG SCID SCID	Subrenal capsule Subrenal capsule Subrenal capsule Subcutaneous	66% 96% 93% 58%	P0 P0 P0 P0	Toivanen et al.^33^ Wetterauer et al.^40^ Wang et al.^64^ Wang et al.^64^
Pancreatic cancer	Nude Nude Nude SCID	Subcutaneous Orthotopic Subcutaneous Subcutaneous	70% 55% 61% 66%	P0 P0 P0 P0	Rubio et al.^77^ Rubio et al.^77^ Garrido et al.^78^ Mattie et al.^79^
Ovarian cancer	Balb/c nude Nude SCID	Subrenal capsule Intraperitoneal Intraperitoneal	49% 31% 74%	P0 P2 P0	Heo et al.^80^ Liu et al.^81^ Karlan et al.^20^
Head and neck cancer	NSG NSG NSG NSG	Subcutaneous Subcutaneous Subcutaneous Subcutaneous	48% 79% 45% 85%	P2 P0 P2 P0	Klingh et al.^21^ Swick et al.^47^ Klingh et al.^82^ Kimple et al.^83^

## Abbreviations

PDX: patient-derived xenograft
NCI: national cancer institute
OS: overall survival
RFS: recurrence-free survival
HER2: human epidermal growth factor receptor 2
PR: progesterone receptor
ER: estrogen receptor
TURP: transurethral resection of the prostate
NSCLC: non-small cell lung carcinoma
SCCHN: squamous cell carcinoma of the head and neck
HRCC: human renal cell carcinoma
CRC: colorectal carcinoma
Hu-PDX: humanized patient-derived xenograft
